# An interesting case of pulmonary hypertension in nephrotic syndrome due to amphetamine use for attention-deficit hyperactivity disorder

**DOI:** 10.1097/MS9.0000000000000355

**Published:** 2023-04-03

**Authors:** Abat Khan, Aamer Ubaid, Muhammad Hanif, Vikash Jaiswal, Ashraf Gohar, Aashna Mehta, Dushyant Ramakrishnan, Abhigan Babu Shrestha

**Affiliations:** a Departments of Internal Medicine; b Respiratory and Critical Care Medicine, University of Missouri Kansas City,Kansas City, MO; c SUNY Upstate Medical University, New York, NY; d AMA School of Medicine, Makati, Philippines; e University of Debrecen-Faculty of Medicine, Debrecen, Hungary; f M Abdur Rahim Medical College, Dinajpur, Bangladesh

**Keywords:** ADHD, amphetamine, end-stage renal disease, minimal change disease, nephrotic syndrome, pulmonary arterial hypertension

## Abstract

Pulmonary arterial hypertension (PAH) was first associated with stimulants use in the 1960s during an outbreak of amphetamine-like appetite suppressants (anorexigens). To date, various drugs and toxins have been correlated with PAH. Diagnosing PAH in nephrotic syndrome has always remained a challenge due to the overlap of signs and symptoms in clinical presentation between the two entities.

**Case presentation:**

In this report, the authors present an interesting case of a 43-year-old male, diagnosed with nephrotic syndrome secondary to minimal change disease, as well as currently presenting with PAH secondary to amphetamine.

**Clinical discussion and conclusion:**

Patients with nephrotic syndrome and end-stage renal disease should be regularly followed up and evaluated for comorbidities, complications, as well as adverse events from pharmacological intervention. In patients with end-stage renal disease hypertension control is key, stimulant use can precipitate poor blood pressure control especially in pulmonary arteries resulting in PAH. PAH can result in right ventricular dysfunction and heart failure that can further exacerbate renal dysfunction and vice-versa in a vicious cycle, deteriorating patient condition and quality of life.

## Introduction

Highlights

Pulmonary hypertension is an adverse effect associated with methamphetamine use.
Patients with nephrotic syndrome should be regularly monitored for kidney function
Any comorbidities and adverse effects of medication should be noted and addressed.

A German physician Klob reported the autopsy finding of an impressive narrowing of the finer branches of the pulmonary artery in a patient who had progressive ankle oedema, cyanosis, and dyspnoea before death in 1865^1^. Later on, in 1891 German physician Romberg described pulmonary arterial hypertension (PAH) as a life-threatening disease that can lead to right ventricular failure and eventually death^1^. Only a few cases of PAH were reported till the 1960s, when a surge in PAH cases were observed secondary to anorexigen use in Austria, Germany, and Switzerland^2^. To address an increasing number of cases of PAH, in 1973 WHO held an international conference, to discuss the current state of knowledge, establish nomenclature and create classification for PAH that remains in use till date^3^. Amphetamine, a FDA-approved drug for treatment of attention-deficit/hyperactivity disorder (ADHD) and narcolepsy^4^, is notorious for causing various biochemical abnormalities and cardiovascular complications^5^,^6^. Hypoglycemia, myoglobinuria on urinalysis, metabolic acidosis on blood gas test, sinus tachycardia, hypertension, tremor, hyperthermia, and myocardial infarction have been reported in the literature due to amphetamine toxicity^5^. Cases of PAH have been reported secondary to amphetamine and its derivatives use in the literature^7^. Only rarely PAH has been reported secondary to amphetamine use in known nephrotic syndrome patients in the literature^8^. Here, we are presenting a 43-year-old male, a known case of nephrotic syndrome secondary to minimal change disease (MCD), diagnosed as PAH secondary to amphetamine.

## Case presentation

A 43-year-old male patient with a past medical history of MCD and ADHD since childhood presented to the emergency department with shortness of breath, generalized oedema, and foamy urine without haematuria for the last few months. The patient was taking Adderall (mixed amphetamine salts with dextroamphetamine and amphetamine) for ADHD since childhood and have undocumented history of inadequately treated MCD. He was evaluated for chronic kidney disease which revealed nephrotic range proteinuria, hypoalbuminemia, and an elevated brain natriuretic peptide. Urine drug sample was positive for amphetamine and cannabinoids. An extensive work-up was negative for secondary causes of nephrotic syndrome and kidney biopsy findings were consistent with MCD.

**Figure 1:**
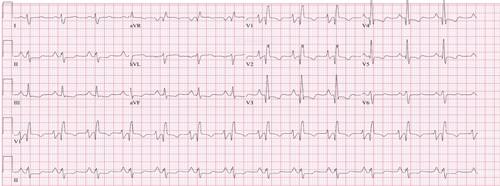
Right bundle branch block pattern observed in the ECG obtained on presentation.

**Figure 2:**
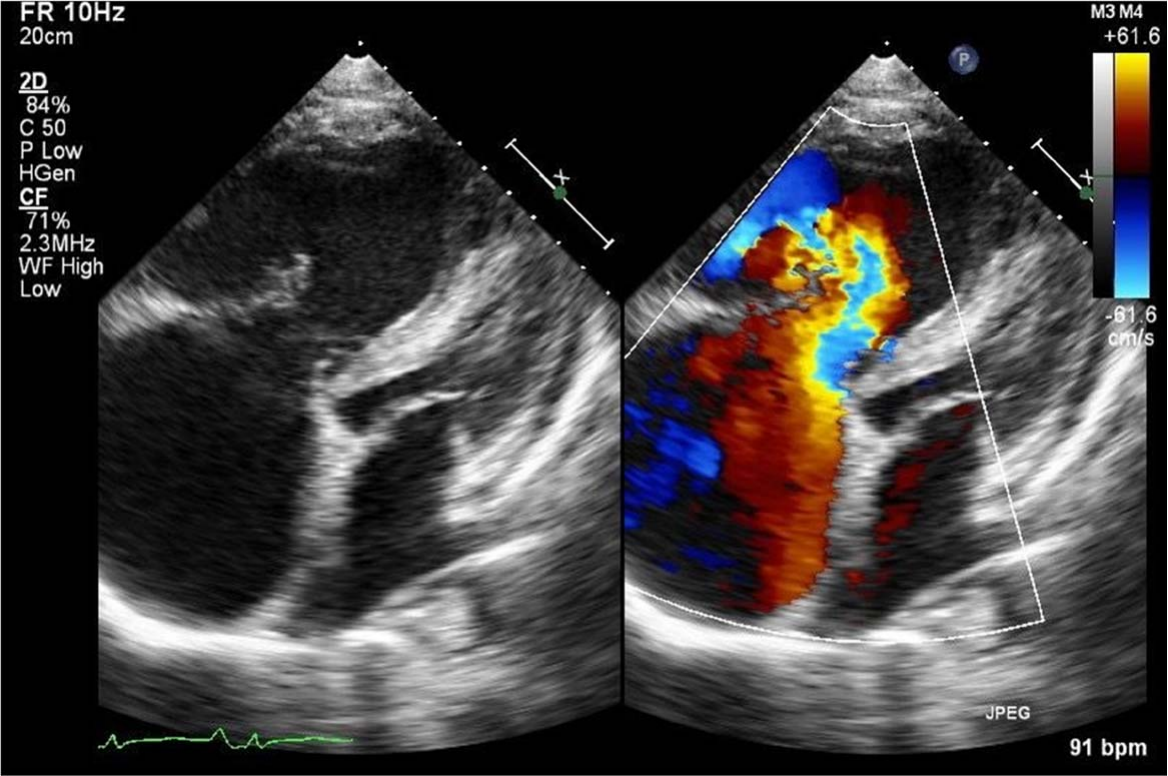
Echocardiographic findings revealing severe dilation of the main right ventricle (RV) and RV outflow tract, significant RV hypertrophy, severely reduced RV systolic function, severe right atrial dilation, severe tricuspid regurgitation, and elevated central pressures.

Electrocardiography was performed that showed right bundle branch block (Fig. 1) and echocardiography (Fig. 2) revealed grade 1 diastolic dysfunction, severe dilation of the main right ventricle (RV) and RV outflow tract, significant RV hypertrophy, severely reduced RV systolic function, severe right atrial dilation, severe tricuspid regurgitation, and elevated central pressures. Chest X-ray showed cardiomegaly with a prominent pulmonary trunk suggestive of pulmonary hypertension. Cardiac catheterization revealed a mean pulmonary artery pressure of 43 mmHg and a pulmonary vascular resistance of 14.3 Wood units, consistent with moderate to severe PH, which was not reversible with the adenosine challenge. The patient commenced on high dose prednisone, lasix, and losartan. Adderall was discontinued as a possible contributing factor to PAH. The patient responded well to the treatment with the improvement in symptoms such as oedema and shortness of breath. Over the next 3 months, a gradual normalization of proteinuria, hypoalbuminemia, and urine protein-to-creatinine ratio was observed.

## Discussion

PAH is characterized by progressive obliteration of pulmonary vasculature, resulting in increased resistance that can lead to right heart failure and ultimately death^9^. PAH can be associated with certain drugs or toxins such as aminorex, fenfluramine derivatives, benfluorex, and rarely with mazindol, phentermine, and amphetamines^9^. Recently a study conducted by Hlavaty A and colleagues, identified 15 new drugs likely to be related to PAH. These drugs were protein kinase inhibitors (lapatinib, lorlatinib, ponatinib, and ruxolitinib), antimetabolites (fludarabine, cytarabine, fluorouacil, and gemcitabine), immunosuppressants (thalidomide, ciclosporin, leflunomide), chemotherapeutics (trastuzumab and etoposide), and angiogenesis inhibitors (bevacizumab)^10^. While the exact pathomechanism by which drugs cause PAH is unclear, it could be explained by increased levels of serotonin in the blood due to interaction between these drugs and serotonin receptors^11^.

Amphetamines cause increased serotonin release, which stimulate proliferation of smooth muscle cells and pulmonary vasoconstriction resulting in pulmonary arterial hypertension^7^. Several studies such as Wang *et al*. 12 found higher concentration of serotonin and higher mean pulmonary arterial pressures in rats that were injected with methamphetamines in comparison to the control group.

The prevalence of pulmonary arterial hypertension is higher in end-stage renal disease (ESRD) and has important prognostic and therapeutic implications. Infact, a study by Schoenberg *et al*.^13^ revealed higher prevalence of pulmonary hypertension in ESRD patients (38%) especially in ESRD patients undergoing haemodialysis (40%) in comparison to those undergoing peritoneal dialysis (19%). The pathophysiology of PAH in CKD patients is multifactorial and includes volume overload with high pulmonary capillary wedge pressure, higher cardiac output secondary to anaemia and an intravenous access to HD patients^14^. Treatment of PAH in a complex case of ESRD should be focused on treating the underlying cause that was stimulant abstinence and possibly suggesting change to other medications to treat comorbidities such as ADHD, managing ESRD and supportive treatment with regular follow-up. Hypertension control is key in managing ESRD, preventing further depreciation in renal function as well as preventing any associated complications such as cardiac dysfunction suggested by the elevated brain natriuretic peptide in this patient. Finally, this report has been made following SCARE guidelines^15^.

PAH has been reported secondary to connective tissue disease in the literature^16^. Minimal change nephrotic syndrome complicating connective tissue order^17^ could lead to causing PAH and this could be one of the possibility of having PAH in our patients but no such symptoms of connective tissue disease were reported in our patient.

## Conclusion

Patients with nephrotic syndrome and ESRD should be regularly followed up and evaluated for comorbidities, complications, as well as adverse events from pharmacological intervention. In patients with ESRD hypertension control is key, stimulant use can precipitate poor blood pressure control especially in pulmonary arteries resulting in PAH. PAH can result in RV dysfunction and heart failure that can further exacerbate renal dysfunction and vice-versa in a vicious cycle, deteriorating patient condition and quality of life. Following clinical guidelines, an evidence-based approach is essential to prescribe and regulate pharmacological drug use in patients with a complex case presentation such as this.

## Ethical approval

Not required.

## Consent from the patients

A written informed consent has been taken from the patient for this case report and images. A copy of which will be made available upon reasonable request from the corresponding author.

## Source of funding

None.

## Author contribution

Substantial contribution to the conception and design of the work: All authors under the guidance of A.K. Drafting the work and critical revision: All authors under the guidance of A.K. and V.J. All the authors read and approved the final version of the manuscript.

## Conflicts of interest disclosure

None declared by the authors.

## Research registration unique identifying number (UIN)

None.

## Guarantor

Dr Abat Khan.

## Provenance and peer review

Not commissioned, external peer-review.
